# Prognostic role of bone erosion in orbital RMS: a report from the European Pediatric Soft Tissue Sarcoma Study Group (EpSSG)

**DOI:** 10.3389/fonc.2024.1497193

**Published:** 2024-12-12

**Authors:** Daniela Di Carlo, Giulia Fichera, Veronique Minard-Colin, Beatrice Coppadoro, Daniel Orbach, Alison Cameron, Monica Albiac Ramos, Myriam Ben Arush, Johannes H. M. Merks, Gianni Bisogno

**Affiliations:** ^1^ Department of Women’s and Children’s Health, University of Padova, Padova, Italy; ^2^ Pediatric Hematology-Oncology Division, University Hospital of Padova, Padova, Italy; ^3^ Pediatric Radiology Unit, University Hospital of Padova, Padova, Italy; ^4^ Department of Pediatric and Adolescent Oncology, Institut national de la santé et de la recherche médicale (INSERM) U1015, Gustave Roussy, Université Paris-Saclay, Villejuif, France; ^5^ Hematology Oncology Division, Department of Women’s and Children’s Health, University of Padova, Padova, Italy; ^6^ Soins, Innovation, Recherche, en oncologie de l'Enfant, de l'aDOlescent et de l'adulte jeune (SIREDO) Oncology Centre (Care, Innovation and Research for Children, Adolescents and Young Adults with Cancer), Pitié Salpêtrière (PSL) University, Institut Curie, Paris, France; ^7^ FRCR–Bristol Haematology and Oncology Centre, University Hospitals Bristol and Weston NHS Foundation Trust, Bristol, United Kingdom; ^8^ Department of Radiation Oncology, Hospital Vall d’Hebron, Barcelona, Spain; ^9^ Joan and Sanford Weill Pediatric Hematology Oncology and Bone Marrow Transplantation Division, Ruth Rappaport Children’s Hospital, Haifa, Israel; ^10^ Princess Máxima Center for Pediatric Oncology, Utrecht, Netherlands; ^11^ Division of Imaging and Oncology, University Medical Center Utrecht, Utrecht University, Utrecht, Netherlands

**Keywords:** orbital tumor, parameningeal, bone erosion, pediatric rhabdomyosarcoma, RMS

## Abstract

**Background:**

Orbital rhabdomyosarcoma (RMS) is often limited to the orbital cavity and has a favorable prognosis. In some cases, the tumor can erode the orbital bone and behave as a parameningeal RMS (PM-RMS); thus, it is treated more intensively. However, the current protocols do not provide any guidance on how to consider different grades of bone erosion (BE) that can vary widely, hampering a uniform classification and the subsequent treatment assignment. With the aim of clarifying the role of BE as a risk factor, we analyzed patients with orbital RMS included in the European Pediatric Soft Tissue Sarcoma Study Group (EpSSG) protocol.

**Methods:**

We retrospectively analyzed the radiological reports of 199 patients with orbital RMS (PM or not) and defined three grades of BE: minimal (thinning of the bone), moderate (focal bone lysis), and extensive (complete cortical destruction).

**Results:**

BE was present in 55 of the 199 (27.6%) patients, which was classified as minimal in 27, moderate in 7, and extensive in 21. Tumors with extensive BE were more frequently large (>5 cm, *p* = 0.0008) and invasive (T2, *p* = 0.001). With a median follow-up of 70.4 months (range = 7.1–167.7), a total of 183 patients are alive, with 5-year event-free survival (EFS) and overall survival (OS) rates of 76% (95%CI = 69.2–81.3) and 92% (95%CI = 86.7–94.8), respectively. Patients without any BE had better OS (95% *vs*. 81%, *p* = 0.001), but not EFS. Patients with no/minimal/moderate BE had better EFS and OS compared with patients with extensive BE [EFS of 78.1 (95%CI = 71.1–83.5) *vs*. 57.1 (95%CI = 33.8–74.9), *p* = 0.0114, respectively, and OS of 94.0 (95%CI = 89.2–96.8) *vs*. 71.1 (95%CI = 46.6–85.9), *p* < 0.0001, respectively]. Events and metastatic relapses (in all cases CNS/meningeal) were more frequent in patients with extensive BE.

**Conclusions:**

Only those patients with orbital RMS and extensive BE should be considered as PM and should be treated accordingly.

## Introduction

Rhabdomyosarcoma (RMS) can develop at any site of the body, and the primary location in the orbit accounts for approximately 10% of patients (1). The tumor site is correlated with prognosis and directs the treatment strategy. Orbit is considered a favorable primary tumor site as around 90% of children with RMS in this location can be cured (2–5). The favorable prognosis may be partly explained by the fact that tumors tend to be limited to the orbital cavity. However, in some patients, the tumor erodes the bone and may extend into the surrounding areas, particularly the intracranial compartment. In European and North American protocols, orbital RMS with bone erosion (BE) is classified as parameningeal RMS (PM-RMS), with a consequent upstaging and the administration of more intensive treatment (5, 6). However, the definition of BE is quite generic since European cooperative groups state that the erosion must be “important,” while the Children’s Oncology Group (COG) requires “invasion of the bone by the tumor.”

The grade of BE may be very different, ranging from minor bone involvement to complete erosion. The current protocols and guidelines do not clearly define BE, potentially leading to differences in interpretation, classification, and treatment.

The aim of this study was to analyze the cohort of patients with orbital RMS (PM or not) included in the European Pediatric Soft Tissue Sarcoma Study Group (EpSSG) RMS 2005 protocol in order to analyze the role of BE as a risk factor.

## Methods

### Population

The EpSSG RMS2005 (EudraCT no. 2005-000217-35) study was conducted from October 2005 to December 2016 and included patients with pathology-proven non-metastatic RMS younger than 21 years. All participating centers were required to obtain written approval from their local authorities and ethics committees and written informed consent from the patient and/or his/her parents or legal guardians. The diagnostic workup included the evaluation of the primary tumor, regional nodes, and possible distant metastases with cranial magnetic resonance imaging (MRI) and/or chest computed tomography (CT), bone scintigraphy, bone marrow aspiration, and biopsy. ^18^F-fluorodeoxyglucose positron emission tomography/computed tomography (^18^F-FDG-PET/CT) was optional; however, if performed, the results were added to the staging of the tumor.

All patients with a primary RMS arising in the orbit with the diagnostic radiological report (cranial MRI scan and/or CT) available in the EpSSG database were included in this analysis. Patients whose reports did not refer to the bone were not included. In the RMS 2005 protocol, the tumor site was defined as orbital when the tumor developed in the orbital cavity and as orbital PM when the tumor arose in the orbital cavity and had an intracranial extension or important BE. The level of BE was not further defined.

The other prognostic risk factors used in the EpSSG stratification were histology, post-surgical stage according to the Intergroup Rhabdomyosarcoma Study (IRS) grouping system, nodal involvement, tumor size (> or <5 cm), and patient age (< or >10 years).

### Radiological reports and bone erosion evaluation

**Figure 1:**
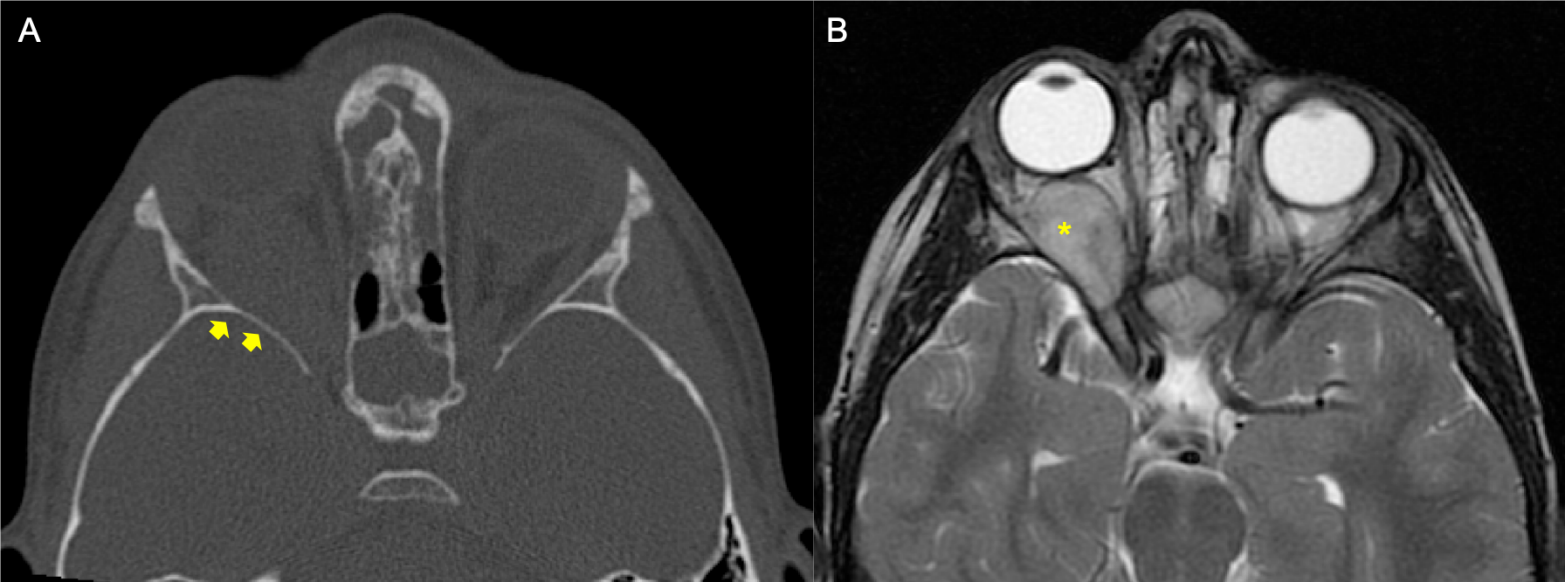
Example of a patient affected by rhabdomyosarcoma (RMS) with no bone erosion (BE). The patient underwent orbital CT scan and MRI at staging. **(A)** Bone window axial reconstruction of the CT did not show BE (*yellow arrows*). **(B)** Axial T2-weighted image of magnetic resonance confirmed right-side orbital mass (*yellow asterisk*).

**Figure 2:**
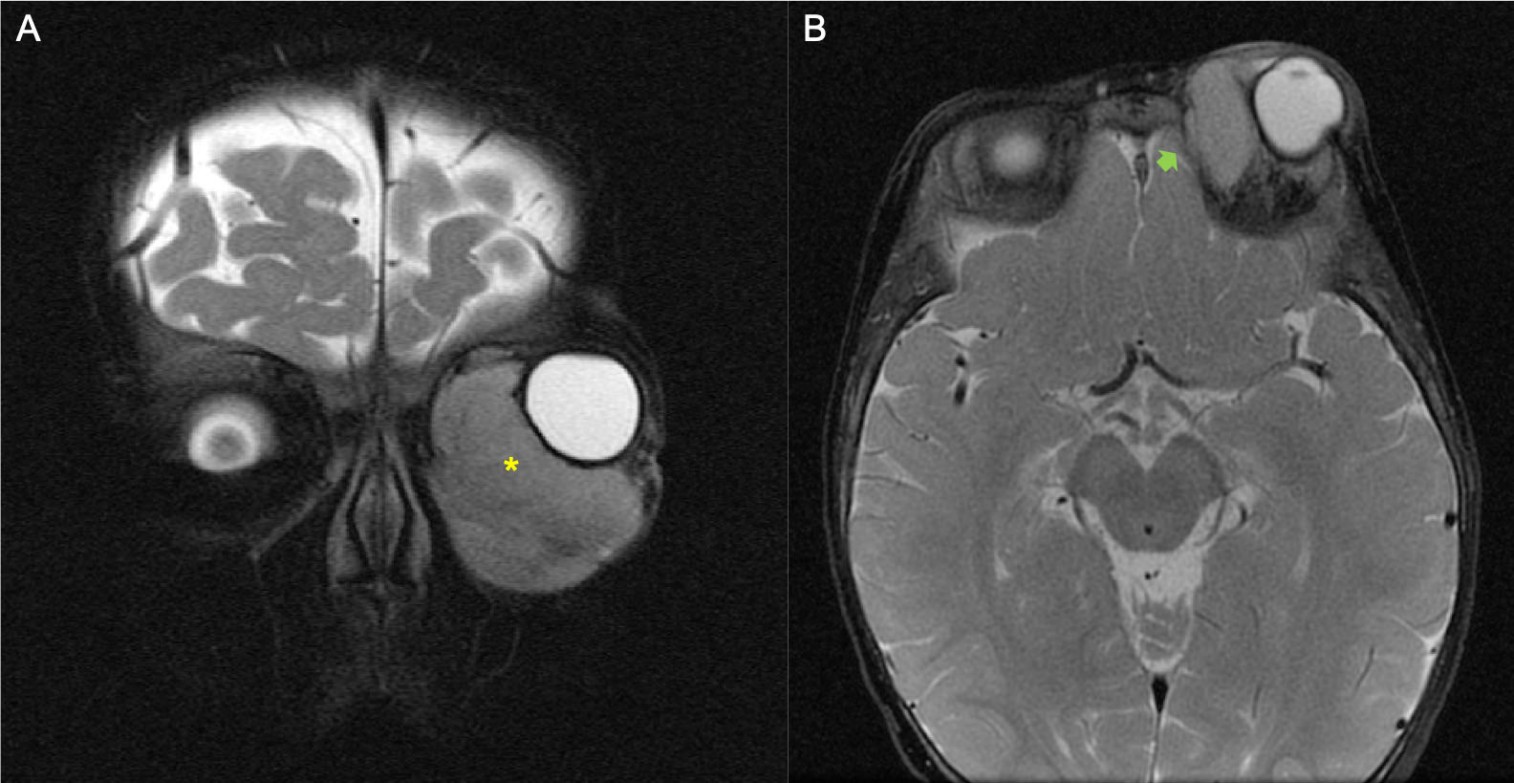
Example of a patient with orbital rhabdomyosarcoma (RMS) and minimal bone erosion (BE). **(A, B)** MRI images showed left orbital tumor on T2-weighted coronal plane (*yellow asterisk*) **(A)** and minimal grade of BE on the axial fat-saturated T2-weighted image (*green arrow*) **(B)**.

**Figure 3:**
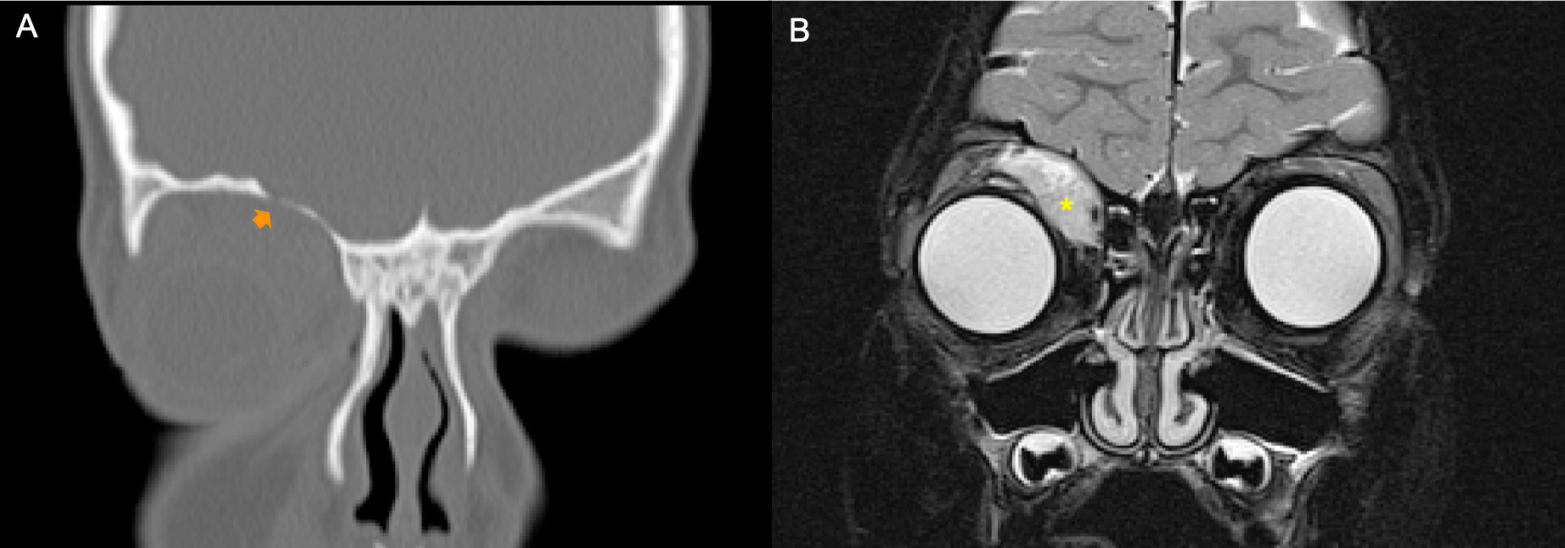
Example of a patient with orbital rhabdomyosarcoma (RMS) and moderate bone erosion (BE). CT scan and MRI were performed at staging. **(A)** Bone window coronal reconstruction of the CT scan showed moderate grade of BE (*orange arrow*). **(B)** Coronal fat-saturated T2-weighted image of magnetic resonance well demonstrated a right orbital tumor (*yellow asterisk*).

**Figure 4:**
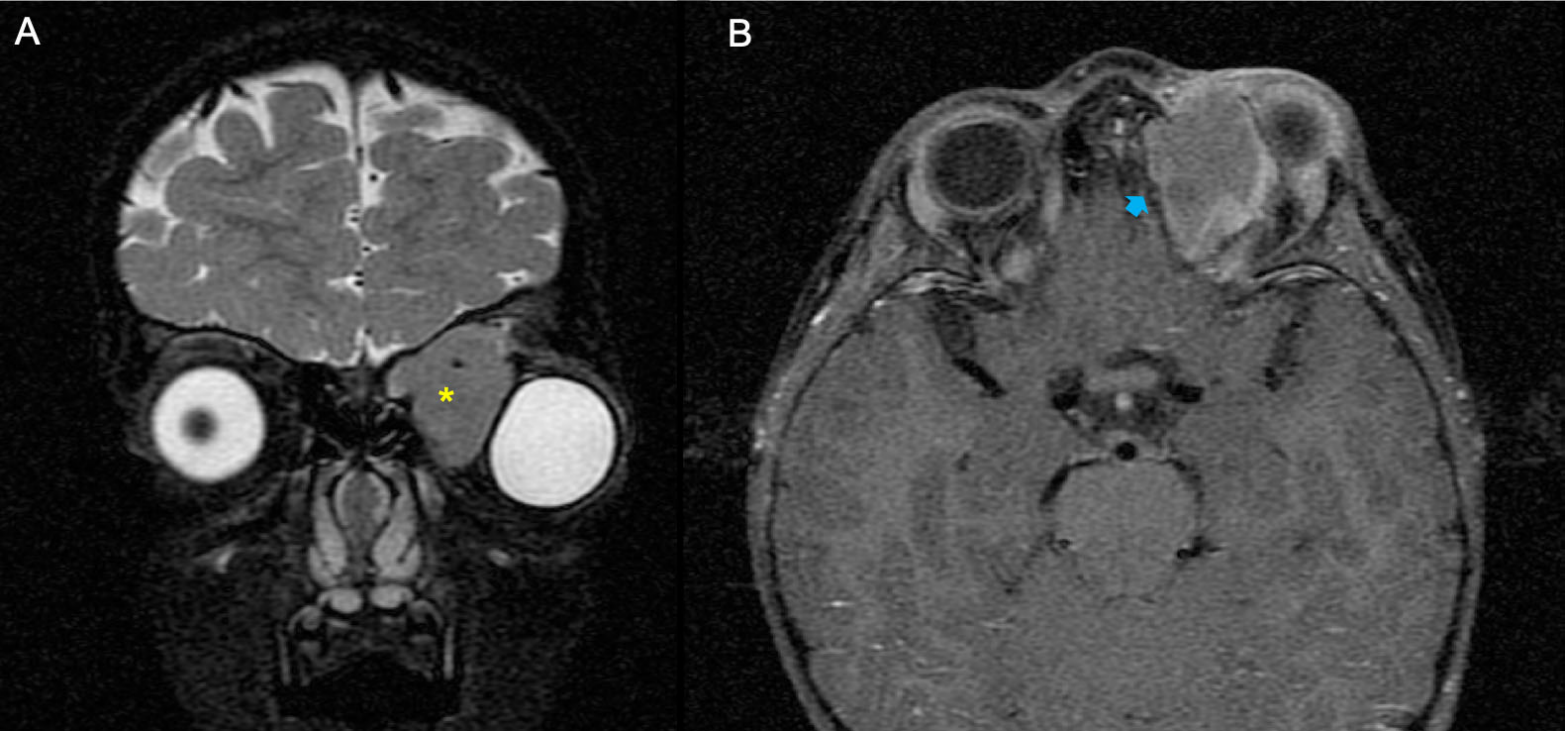
Example of a patient with orbital rhabdomyosarcoma (RMS) and extensive bone erosion (BE). **(A, B)** MRI showed left orbital mass on the coronal plane fat-saturated T2-weighted image (*yellow asterisk*) **(A)** and an extensive grade of BE on the axial T1 fat-saturated post-contrast sequence (*blue arrow*) **(B)**.

The MRI and CT scan performed at the time of diagnosis were independently reviewed by two authors (DDC, oncologist, and GF, pediatric radiologist). Orbital RMS were initially reclassified into two groups, i.e., tumors with BE and those without BE. Where BE was described, it was graded. Without a validated grading system, three levels of BE were defined: minimal, moderate, and extensive (**Figures 1**–**4**). BE was defined as minimal when the report described scalloping, remodeling, and/or thinning of the bone caused by the tumor; BE was moderate when focal bone lysis or focal cortical interruption was described; and BE was considered as extensive in the case of complete cortical destruction, with the tumor crossing the preexisting bone barrier (**Figures 1**–**4**). The presence of central nervous system (CNS) involvement was also annotated.

### Treatment

The treatment strategy recommended in the RMS 2005 protocol has been previously described. The chemotherapy regimens according to the risk group are summarized in **Supplementary Table S1** (7–10).

In brief, for orbital and PM tumors, the treatment was based mainly on chemotherapy and radiotherapy (RT), with a biopsy recommended for the initial diagnosis. Delayed surgery was not encouraged due to the difficulties of performing a complete conservative resection in this specific body area. Patients with orbital embryonal RMS were assigned to subgroup C, and chemotherapy included the administration of four cycles of IVA (3 g/m^2^ ifosfamide, on days 1 and 2; 1.5 mg/m^2^ vincristine, given as a single i.v. injection on day 1; and 1.5 mg/m^2^ actinomycin D, on day 1) followed by five cycles of VA. In the absence of irradiation (which was optional if there was a complete response to chemotherapy at week 9), the patients received five additional cycles of IVA instead of VA. Patients with PM tumors were allocated to the standard risk subgroup D, for those <10 years with small tumors (<5 cm), or to the high-risk group, for older patients or those with larger tumors. The patients in subgroup D received nine cycles of IVA, while high-risk patients were randomized to either nine IVA courses or four IVADo courses (IVA combined with doxorubicin) followed by five IVA courses (8). These patients were also eligible to receive maintenance chemotherapy randomly with 6 months of vinorelbine and oral low-dose cyclophosphamide (9).

All patients with alveolar RMS and nodal involvement were assigned to the very high-risk group and received four IVADo courses followed by five IVA courses and maintenance treatment. Tumor response assessment was scheduled after three courses applying the same modalities used at diagnosis. It was defined as follows: complete remission (CR) in the case of complete disappearance of the lesion; partial response (PR) in the case of regression between 66% and 89%, for volume reduction >1/3; progression of disease (PD) in the case of a 40% increase in the tumor volume or evidence of new lesions; and stable disease (SD) if no criteria for PR or PD.

Radiotherapy commenced in week 13, with a dose that was dependent on the tumor histology, chemotherapy response, and the IRS group, and ranged between 36 and 55.8 Gy. For patients with embryonal orbital RMS and a complete response, there was an option between omitting RT or radiation to the whole orbit to 36 Gy and then boosting to the primary site to a total of 41.4 Gy. Otherwise, in embryonal orbital RMS, after whole orbit radiation, the boost was to a total dose of 45 Gy after PR and 50.4 Gy for the rest. In embryonal PM and alveolar orbital or PM-RMS, the RT field covered the whole orbit to 36 Gy plus all areas of initially involved disease to 41.4 Gy and, unless embryonal with complete response or complete resection, a boost dose to 50.4–55.8 Gy. However, the intracranial component was only irradiated if it was still present at week 9 reassessment. Regarding surgery, R classification was used to consider the clinical and pathological findings. R0 corresponds to resection for cure or CR, R1 to microscopic residual tumor, and R2 to macroscopic residual tumor (11).

### Statistical methods

Survival curves were calculated with the Kaplan–Meier method: overall survival (OS) was estimated from diagnosis to death due to any cause or the last follow-up. Event-free survival (EFS) was calculated from diagnosis to the first tumor event or second tumors. The log-rank test was used to compare the survival curves of patient subgroups on univariate analysis to ascertain the potential value of the prognostic factors. These factors were included in a multivariable analysis. A stepwise selection was applied to select the most significant variables.

## Results

### Population

Overall, 219 patients with an RMS arising in the orbit were considered for this study. We excluded eight patients without available radiological reports and 12 patients whose reports were available but without any bony reference. Therefore, 199 patients were included in the analysis. The clinical characteristics of the patients are reported in **Table 1**. Of the 199 patients, MRI reports were available for 162, CT scan reports for 115, and both MRI and CT scans for 78. In none of the cases was there a discordance between the MRI and CT scan concerning BE.

**Table 1:** Description of the characteristics of the patients and of the tumor by bone erosion (BE).

	No BE (*N* = 144)	Yes BE (*N* = 55)	Total no. of patients	Comparison with *p*-value
Age at diagnosis				
≤1 year	1	1	2	*0.2259*
1–9 years	107	42	149	
10–17 years	35	10	45	
≥18 years	1	2	3	
Median age, years (range)	*6.6 (0.7–19.6)*	*6.2 (0.8–21.8)*	*6.6 (8.0 months–21.8 years)*	
Gender				
Female	55	26	81	0.2437
Male	89	29	118	
Histology				
Favorable RMS	129	42	171	0.0165
Unfavorable RMS	15	13	28	
Fusion status				
Negative	102	33	135	0.2354^a^
Positive	5	4	9	
Not available	37	18	55	
Site (as per enrolment)				
Orbit	141	28	169	*<0.0001*
Orbital primary classified as PM	3	27	30	
Orbit location				
Whole orbit	6	1	7	*0.0174*
Lower area	15	15	30	
Upper area	80	19	99	
Lateral	13	5	18	
Medial	24	14	38	
Not specified	6	1	7	
T-invasiveness				
T1	125	21	146	*<0.0001*
T2	19	34	53	
Tumor primary size				
≤5 cm	136	44	180	*0.0012* ^b^
>5 cm	5	10	15	
Not available	3	1	4	
Nodal involvement				
N0	141	54	195	0.5637^c^
N1	3	–	3	
N*x*	–	1	1	
IRS group				
IRS I	1	–	1	0.5334
IRS II	22	5	27	
IRS III	121	50	171	

Loco-regional lymph nodes site: 2, cervical; 1, cervical, parotideal, pre-auricular, or jugular. Chi-square test and *Fisher’s exact test* were used to compare the variables.

a Only patients with the fusion status available were considered.

b Patients with size *x* were excluded.

c Patients with N*x* were excluded.p-values are indicated in Italic that means statistically significant.

Overall, of the 199 patients, 169 were registered in the RMS 2005 study as orbital and 30 as orbital PM-RMS.

The review identified BE in 55 patients (27.6%): 28 orbital, with extensive BE in 4 cases, moderate in 4, and minimal in 20; 27 orbital PM, with extensive BE in 17 patients, moderate in 3, and minimal in 7.

Patients with BE had a significantly higher prevalence of alveolar histology (*p* = 0.009), extension to PM areas (*p* < 0.001), primary involvement of the upper and lower areas of the orbit (*p* = 0.001), and tumors >5 cm (*p* = 0.0012). Considering the grade of BE, patients with extensive BE had more frequent extension to the PM area (*p* = 0.0004), tumor >5 cm (*p* = 0.0008), and T2 invasiveness (*p* = 0.0019) compared with the minimal or moderate erosion subgroups.

### Treatment

Overall, of the 199 patients, 148 (74%) underwent a biopsy as the initial procedure, while 51 (26%) had surgical resection of the primary tumor (1 R0, 27 R1, and 23 R2).

Patients received chemotherapy according to the risk group. Of the 199 patients, the IVA/VA regimen was adopted in 103 (52%), IVA in 79 (40%), and IVADo/IVA in 17 (8%).

Radiotherapy was administered to 175 of the 199 (88%) patients. The reasons for not performing RT included age <3 years (*n* = 3), center decision (*n* = 14), early progression of disease (*n* = 1), and unknown reasons (*n* = 6). Only 4 out of 55 patients with BE did not receive RT. None had intracranial extension, three had minimal BE, and one had moderate BE (patient with surgery that resulted in no tumor found, decision of the center not to irradiate).

### Outcome

With a median follow-up of 70.4 months (range = 7.1–167.7), 151 patients are alive in first CR, while 48 patients had an event. The events were local recurrence (LR) in 33 (68%), metastasis in 9 (18%), combined (LR + metastasis) in 3 (6%), and PD in 1 (2%).

With regard to the 48 patients who had an event, 16 (33%) had initial BE (nine extensive, one moderate, and six minimal). The presence of BE alone was not associated with a higher event rate, but patients with BE had more metastatic events [50% (8/16) in the group with BE *vs*. 12% (4/32) in the group without BE, *p* = 0.005]. Moreover, in all cases, metastasis was located in the CNS (brain or leptomeningeal).

In more detail, in 33 patients with LR, 8 (25%) had BE (five of them had RT) and 25 (75%) had no BE (13 of them had RT). According to our data, there was no difference in the distribution of patients with/without BE and the administration of RT.

Among 12 patients with a metastatic or combined event, nine presented CNS relapse with leptomeningeal dissemination, 6 (67%) of whom had BE (four extensive and two minimal) and 8 (89%) received RT.

Two patients (4%) developed a second tumor (a low-grade glioma in a patient with type 1 neurofibromatosis and a new RMS that was considered as a second tumor by the treating physician).

Moreover, with regard to the 21 patients identified with extensive BE, nine had an event; in six cases, the event was a metastatic relapse (CNS/leptomeningeal). Overall, 32 out of 48 patients with an event were alive at the last follow-up.

**Figure 5:**
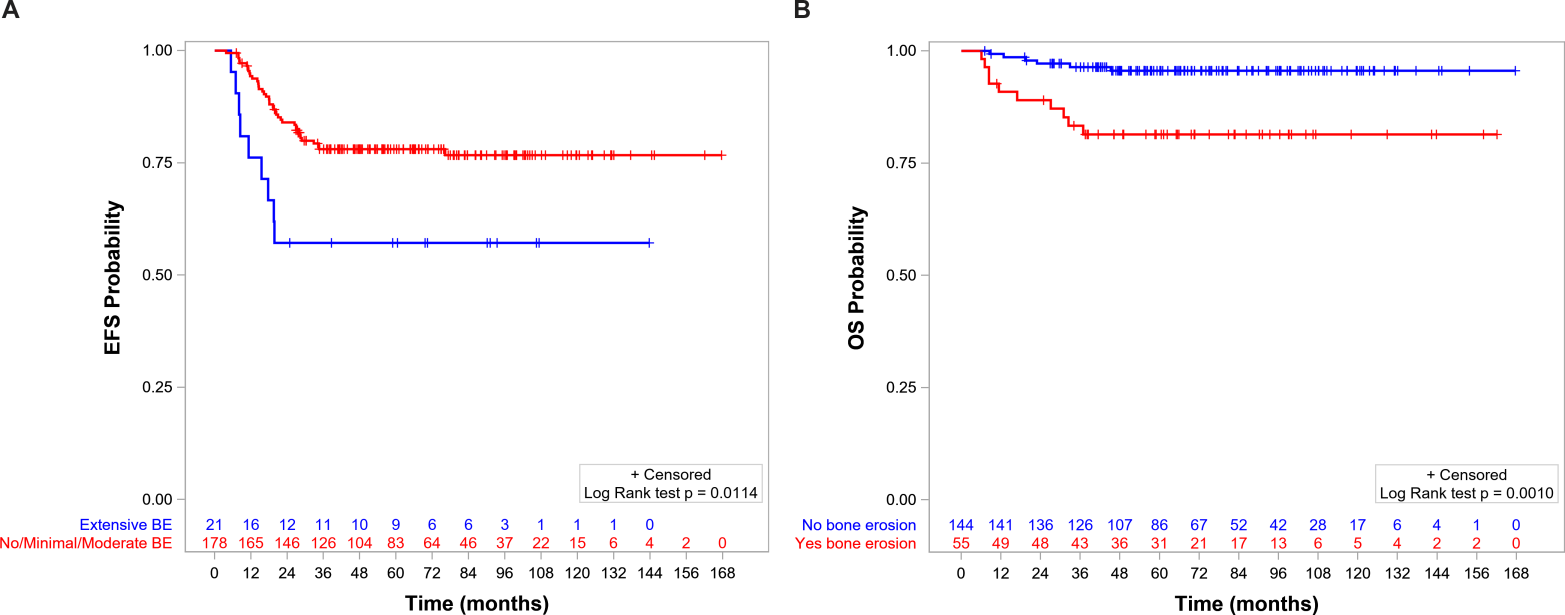
Event-free survival **(A)** and overall survival **(B)** by bone erosion.

**Figure 6:**
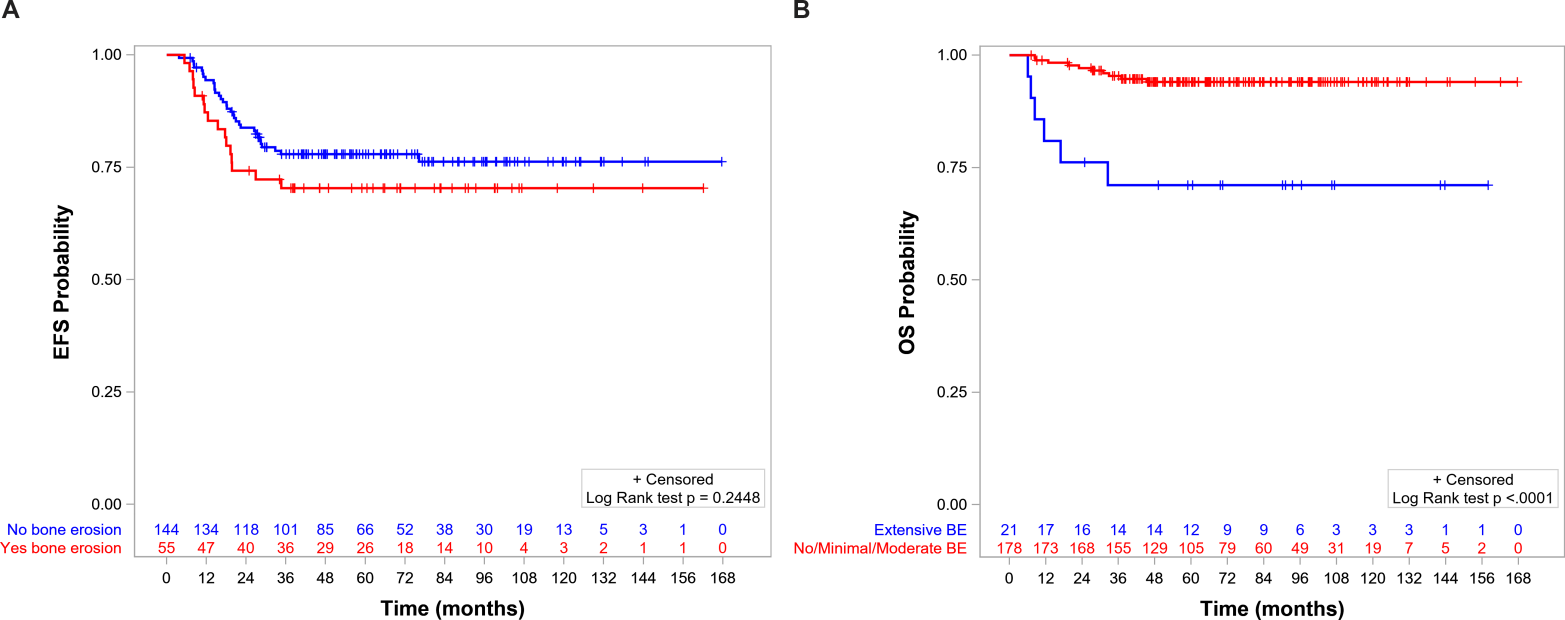
**(A)** Event-free survival (EFS) by level of bone erosion (BE): extensive (5-year EFS = 78.1%, 95%CI = 71.1–83.5) *versus* no/minimal/moderate (5-year EFS = 57.1%, 95%CI = 33.8–74.9, *p* = 0.01). **(B)** Overall survival (OS) by level of BE: extensive (5-year OS = 94%, 95%CI = 89.2–96.8) *versus* no/minimal/moderate (5-year OS = 71.1%, 95%CI = 46.6–85.9, *p* < 0.0001).

The 5-year EFS and OS for the whole population were 75.8% (95%CI = 69.2–81.3) and 91.6% (95%CI = 86.7–94.8), respectively. OS, but not EFS, was significantly lower in patients with BE than in those without BE (**Figures 5A, B**, respectively). EFS and OS were not statistically different when comparing patients with no, minimal, and moderate BE (**Supplementary Figures S1**, **S2**). The group of patients with no/minimal/moderate BE together had better EFS and OS compared with the patients with extensive BE: EFS of 78.1 (95%CI = 71.1–83.5) *vs*. 57.1 (95%CI = 33.8–74.9, *p* = 0.0114) (**Figure 6A**), respectively, and OS of 94.0 (95%CI = 89.2–96.8) *vs*. 71.1 (95%CI = 46.6–85.9, *p* < 0.0001), respectively (**Figure 6B**).

In the multivariable analysis, none of the variables included in the model appears to have a significant effect on EFS. In contrast, for OS, the only significant factor was tumor invasiveness, with a hazard ratio of T2 with respect to T1 of 7.85 (95%CI = 2.46–25.05, *p* = 0.0005).

## Discussion

Patients with orbital RMS have a good prognosis and are treated according to modern protocols based on the administration of relatively intensive chemotherapy and RT; around 90% of these patients are long-term survivors (12). Our analysis confirmed this, where patients with orbital RMS showed a 5-year OS of 91.6%. These overall satisfactory results allowed cooperative groups to reduce the intensity of chemotherapy, and, at present, most children included in COG and EpSSG protocols are treated without or with a limited cumulative dose of alkylating agents, respectively (8, 13). Patients with PM-RMS have a less favorable prognosis, with a 5-year OS of approximately 70%, and therefore need more effective treatment (6, 14).

International protocols currently recommend considering patients with RMS arising in the orbit and with evidence of BE as PM. This implicates upstaging of the tumor and, consequently, a more intensive treatment. However, the grade of BE and its influence on prognosis have never been defined.

In an attempt to analyze the role of BE, we proposed a grading system based on the radiological extension of bone involvement. A preliminary literature search identified limited data. In 1987, researchers from the Memorial Sloan Kettering Center found that extensive BE influenced the outcome and local control of disease in patients with head and neck non-orbital RMS. Extensive BE was defined as the erosion of multiple areas and/or the base of the skull, but they did not consider patients with less extensive BE (15).

Outside RMS, in nasopharyngeal cancer, a different classification was used: BE was defined as type A when intensive bone destruction was present, through varying degrees of intracranial invasion with CNS involvement to subtle bone defects without CNS involvement; type B when only bone sclerosis and minimal signs of erosion were seen; and type C when both A and B were present. Patients with local skull base bone destruction (type A) diagnosed by CT had a significantly increased risk of distant bone metastases in nasopharyngeal carcinoma, N0 or N1 (16).

The system we propose here allows classifying patients into three categories and showed that patients with minimal signs of bone alterations, such as scalloping, thinning, and focal interruptions, appear to share the same favorable prognosis as patients without BE.

For the purpose of this analysis, patients who received different chemo regimens were compared, as outlined in the results. In any case, most patients received the IVA/VA or IVA regimen, and as recently published, the addition of alkylating agents did not show an improvement in terms of survival (17). Moreover, only 8% of the patients received doxorubicin in addition to the IVA regimen according to their risk group, and this addition did not improve the outcome of patients either (8).

The children with extensive BE presented a higher event rate and a worse prognosis. Interestingly, these patients had a high risk of CNS leptomeningeal relapse, which could explain why the OS and EFS were significantly lower in the group with extensive BE. Patients with metastatic relapse, and especially with leptomeningeal extension, are extremely difficult to be salvaged (18). RT fields generally treat the tumor bed as it was at the initial presentation, plus a margin for subclinical spread. However, the EpSSG RMS 2005 guidelines recommended only treating intracranial disease if this was still present at the time of reassessment in order to limit the neurocognitive effects of cranial irradiation, which may have impacted the risk of intracranial relapse. The guidelines for the current EpSSG FaR-RMS study recommend treating the extension of disease at the time of initial presentation (including intracranial), which may help limit CNS relapse. However, other options, including chemotherapy and the timeliness of radiation, should also be considered (19).

Analyzing the radiological reports of patients with orbital primary RMS enrolled in the EpSSG RMS 2005 protocol, we identified the presence of BE in 27% of the patients. According to the RMS 2005 recommendations, patients had to be considered and treated as PM-RMS when important BE was present. The decision regarding the meaning of “important bone erosion” is left to the treating physician. If we assume that “important” means extensive erosion according to our definition, then 25% of patients with BE have been misclassified as three cases of moderate BE and seven cases with minimal BE have been considered as PM; on the other hand, four patients with extensive BE have been classified as orbital RMS.

A central radiological review was not conducted in the EpSSG 2005 study, which may have led to variability in the reporting and the interpretation of the staging scans across different centers. This study relied on written radiological reports from a broad geographic range, introducing potential inconsistencies in data interpretation. As such, the lack of a centralized radiological review represents a significant limitation of this study.

Unfortunately, we were not able to evaluate the RT plan of the patients included in our analysis to determine whether the relapse occurred within or outside the radiation field. However, all patients enrolled in the ongoing EpSSG Frontline and Relapse-Rhabdomyosarcoma (FaR RMS) trial (EudraCT no. 2018-000515-24, NCT04625907) have RT quality assurance of contouring and the RT plan to improve adherence to the RT guidelines, as well as facilitating future analysis (20). In addition, the limited number of patients precluded us from the possibility of making more specific sub-analyses, and multivariate analyses were not able to identify independent prognostic risk factors.

In consideration of the retrospective nature of this trial, the impossibility of reviewing radiological images, and the limited number of patients, a prospective study to validate the system we proposed is necessary.

In conclusion, this retrospective analysis underlines that patients with minimal and moderate BE share the same behavior as those without any BE and should be considered in the same group. Conversely, patients with extensive BE have poorer outcomes and more events. However, the absence of image reviews and the retrospective nature of this analysis prevented us from giving strong conclusions. Therefore, a prospective analysis is needed.

## Data Availability

The original contributions presented in the study are included in the article/**Supplementary Material**. Further inquiries can be directed to the corresponding author.
