# Supplementary material for: Prognostic role of bone erosion in orbital RMS: a report from the European Pediatric Soft Tissue Sarcoma Study Group (EpSSG)
Source: Front Oncol. 2024 Dec 12;14:1497193. doi: 10.3389/fonc.2024.1497193 (PMC11669659; doi:10.3389/fonc.2024.1497193)
Supplement: Supplementary file 1 [file DataSheet1.docx]

Supplementary Material

| **Risk group** | **Sub groups** | **Pathology** | **Post-surgical stage (IRS group)** | **Site** | **Node stage** | **Size and age** | **Chemotherapy** | **Delayed surgery** | **Radiation therapy** |
| --- | --- | --- | --- | --- | --- | --- | --- | --- | --- |
| Low | A | Favorable | I | Any | N0 | Favorable | 8IVA | Not necessary | No |
| Standard Risk | B | Favorable | I | Any | N0 | Unfavorable | 4IVA + 5VA | Not necessary | No |
|  | C | Favorable | II, III | Favorable | N0 | Any | 9IVA or 5IVA +4VA if RT | Yes, if not mutilating | Optional |
|  | D | Favorable | II, III | Unfavorable | N0 | Favorable | 9IVA | Yes, if not mutilating | Yes |
| High Risk | E | Favorable | II, III | Unfavorable | N0 | Unfavorable | 9IVA vs 4IVADo + 5IVA +/-6 maintenance | Yes | Yes |
|  | F | Favorable | II, III | Any | N1 | Any |  |  | Yes |
|  | G | Unfavorable | I, II, III | Any | N0 | Any |  |  | Yes |
| Very High Risk | H | Unfavorable | II, III | Any | N1 | Any | 4IVADo + 5IVA +/-6 maintenance | Yes | Yes |

**Supplementary Table 1**. Risk grouping stratification and therapy in EpSSG RMS 2005 study. Pathology (histology): Favorable = all embryonal, spindle cells, botryoid RMS. Unfavorable = all alveolar RMS (including the solid-alveolar variant). Post-surgical stage (according to the IRS grouping, see appendix A.2): Group I = primary complete resection (R0); Group II = microscopic residual (R1) or primary complete resection but N1; Group III = macroscopic residual (R2). Site = Favorable: orbit, GU non-bladder prostate (i.e. paratesticular and vagina/uterus) and non-PM Head & neck. Unfavorable = all other sites (parameningeal, extremities, GU bladder prostate and ‘other site’). Node stage (According to the TNM classification, see appendix A1 and A.5): N0 = no clinical or pathological node involvement. N1= clinical or pathological nodal involvement. Size and age: Favorable = Tumor size (maximum dimension) 5 cm and age <10 years. Unfavorable = all others (i.e. size >5 cm or age 10 years). Chemotherapy: VA= vincristine-dactinomycin; IVA= ifosfamide-vincristine-dactinomycin; IVADo = IVA-Doxorubin. (from Gosli et al.) (9)

**Supplementary Figure 1.** EFS probability by minimal/moderate vs no BE.


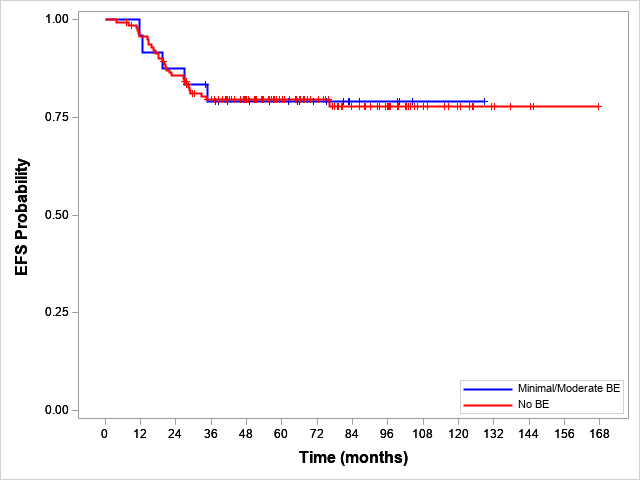


|  | **N** | **Events** | **5-yr EFS (95%CI)** | **p-value** |
| --- | --- | --- | --- | --- |
| No bone erosion | 141 | 29 | 79.6 (71.8-85.4) | 0.9720 |
| Minimal/Moderate BE | 24 | 5 | 78.9 (56.6-90.7) |  |

**Supplementary Figure 2.** OS probability by minimal/moderate vs no BE.


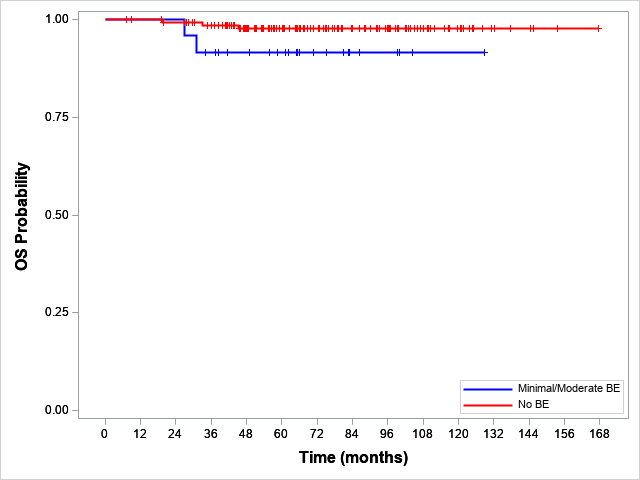


|  | **N** | **Deaths** | **5-yr OS (95%CI)** | **p-value** |
| --- | --- | --- | --- | --- |
| No bone erosion | 141 | 3 | 97.6 (92.8-99.2) | 0.1103 |
| Minimal/Moderate BE | 24 | 2 | 91.7 (70.6-97.8) |  |
